# Characteristics and Timing of Initial Virus Shedding in Severe Acute Respiratory Syndrome Coronavirus 2, Utah, USA

**DOI:** 10.3201/eid2702.203517

**Published:** 2021-02

**Authors:** Nathaniel M. Lewis, Lindsey M. Duca, Perrine Marcenac, Elizabeth A. Dietrich, Christopher J. Gregory, Victoria L. Fields, Michelle M. Banks, Jared R. Rispens, Aron Hall, Jennifer L. Harcourt, Azaibi Tamin, Sarah Willardson, Tair Kiphibane, Kimberly Christensen, Angela C. Dunn, Jacqueline E. Tate, Scott Nabity, Almea M. Matanock, Hannah L. Kirking

**Affiliations:** Centers for Disease Control and Prevention, Atlanta, Georgia, USA (N.M. Lewis, L.M. Duca, P. Marcenac, E.A. Dietrich, C.J. Gregory, V.L. Fields, M.M. Banks, J.R. Rispens, A. Hall, J.L. Harcourt, A. Tamin, J.E. Tate, S. Nabity, A.M. Matanock, H.L. Kirking);; Utah Department of Health, Salt Lake City, Utah, USA (N.M. Lewis, K. Christensen, A.C. Dunn);; Davis County Health Department, Clearfield, Utah, USA (S. Willardson);; Salt Lake County Health Department, Salt Lake City (T. Kiphibane)

**Keywords:** COVID-19, coronavirus disease, SARS virus, virus shedding, severe acute respiratory syndrome coronavirus 2, SARS-CoV-2, viruses, respiratory infections, zoonoses, reverse transcription PCR

## Abstract

Virus shedding in severe acute respiratory syndrome coronavirus 2 (SARS-CoV-2) can occur before onset of symptoms; less is known about symptom progression or infectiousness associated with initiation of viral shedding. We investigated household transmission in 5 households with daily specimen collection for 5 consecutive days starting a median of 4 days after symptom onset in index patients. Seven contacts across 2 households implementing no precautionary measures were infected. Of these 7, 2 tested positive for SARS-CoV-2 by reverse transcription PCR on day 3 of 5. Both had mild, nonspecific symptoms for 1–3 days preceding the first positive test. SARS-CoV-2 was cultured from the fourth-day specimen in 1 patient and from the fourth- and fifth-day specimens in the other. We also describe infection control measures taken in the households that had no transmission. Persons exposed to SARS-CoV-2 should self-isolate, including from household contacts, wear a mask, practice hand hygiene, and seek testing promptly.

The coronavirus disease (COVID-19) outbreak first recognized in Wuhan, China, in December 2019 is now a global pandemic (*1*). Serial intervals for transmission have been estimated (*2*,*3*), and presymptomatic transmission from confirmed case-patients to others has been documented (*4*–*8*). In addition, studies suggest that virus shedding can begin before the onset of symptoms (*7*,*8*) and extend beyond the resolution of symptoms (*9*). However, data on the initiation and progression of viral shedding in relation to symptom onset and infectiousness are limited. Intensive early monitoring of household members through serial (i.e., daily) collection of a respiratory tract specimen for testing by real-time reverse transcription PCR (rRT-PCR), which could clarify the characteristics of initial viral shedding, has rarely been implemented, although serial self-collection of nasal and saliva samples was used in a recent study (*10*). To examine the transmission dynamics of severe acute respiratory syndrome coronavirus 2 (SARS-CoV-2) and guide public health recommendations, we describe initial detection and progression of SARS-CoV-2 viral shedding, as indicated by rRT-PCR positivity for SARS-CoV-2 and cycle threshold (C_t_) values, in relation to exposure to an index patient, symptom onset and duration, and transmission to household contacts who underwent intensive early monitoring with viral cultures.

## Methods

Index patients with laboratory-confirmed SARS-CoV-2 infection were reported to 2 health departments in the Salt Lake City, Utah, USA, metropolitan area during April 19–25, 2020. Households were recruited through convenience sampling with assistance from health department staff and were considered eligible if the index patient was not hospitalized, lived with >2 additional persons, and tested positive for SARS-CoV-2 by rRT-PCR in a respiratory tract specimen collected <5 days before enrollment. A sample size of 5 households was chosen because of time constraints and workload capacity; we also took into consideration the likelihood of observing secondary transmission within households, on the basis of the estimated secondary attack rate in a larger household transmission investigation conducted by the Centers for Disease Control and Prevention (CDC) (*11*). CDC investigation staff visited all enrolled households (day 0) within 2–4 days of diagnosis (within 3–5 days of symptom onset) and conducted daily visits on 4 subsequent days (days 1‒4) and a final visit on day 14.

Before the day 0 visit, questionnaires were administered to all index patients and household contacts by telephone to request demographic information and data on symptoms, exposure to the index patient and others outside the household, and any previous SARS-CoV-2 testing. A household-level questionnaire, completed by the index patient or self-declared head of household, documented the home’s square footage; the number of persons per bedroom and bathroom; isolation measures undertaken by the index patient; and extent of household use of gloves, masks, or cloth face coverings after symptom onset in the index patient. A household-level closeout questionnaire reassessing isolation measures and glove and face mask use during the observation period was completed on the day 14 visit. In addition, during the day 0 and day 14 visits, nasopharyngeal swab specimens and blood samples were collected from all index patients and household contacts. During day 1–4 follow-up visits, nasopharyngeal swab specimens were collected daily from non–index patient household members, including those with SARS-CoV-2 test results pending or confirmed from specimens collected at other facilities before the investigation. If symptoms occurred in a household contact during days 1–14 that were not reported on day 0, investigation staff conducted an interim household visit, during which nasopharyngeal swab specimens were collected from all household members, including the index patient.

During days 1–4, if a household contact had an inconclusive result (1 of 2 target gene regions positive for SARS-CoV-2 by rRT-PCR assay) or positive result (both target gene regions positive) after an rRT-PCR–negative test (i.e., first detection of viral shedding), the associated specimen and all subsequent daily specimens from the person were submitted for viral culture to evaluate infectiousness. Results that were inconclusive by rRT-PCR were categorized as negative unless a positive viral culture was obtained from the same specimen. Specimens positive by rRT-PCR that were collected on day 14 with C_t_ values <35 were also cultured. For household contacts, the date of first positive test was defined as the day on which the first SARS-CoV-2–positive specimen was collected. The Utah Public Health Laboratory (UPHL) tested specimens by using the CDC 2019 novel coronavirus (2019-nCoV) real-time RT-PCR assay (*12*); viral cultures were performed at CDC (*13*). Nasopharyngeal specimens were transported at 4^○^C in viral transport media, first from households to UPHL and then (if applicable) onward to CDC for viral culture. Blood samples were processed by UPHL; serum samples were subsequently shipped to CDC and tested by using a CDC-developed SARS-CoV-2 ELISA kit (B. Freeman, unpub. data, https://doi.org/10.1101/2020.04.24.057323).

During days 0–14, all index patients and household members completed a daily symptom diary. Symptoms were grouped according to Council of State and Territorial Epidemiologists (CSTE) categories of classic (cough, shortness of breath, or discomfort while breathing), nonclassic (>2 of measured or subjective fever, chills, headache, myalgia, sore throat, loss of taste, or loss of smell), and asyndromic (symptoms other than CSTE classic or nonclassic) (*14*). Symptom onset was defined as the first day of any reported symptom. Onset of viral shedding was defined as the date of first detection of SARS-CoV-2 by rRT-PCR in the nasopharynx. Presymptomatic shedding was defined as symptom onset >1 day after the first positive SARS-CoV-2 result by rRT-PCR. C_t_ values were categorized as low (<20), medium (20–30), and high (>30). Lower C_t_ values indicated that more viral RNA was detected in the specimen.

This protocol was reviewed by CDC human subjects research officials, and the activity was deemed nonresearch as part of the COVID-19 public health response. Verbal assent to participate was initially obtained by telephone during questionnaire administration, and written consent was collected during the first visit.

## Results

**Figure 1:**
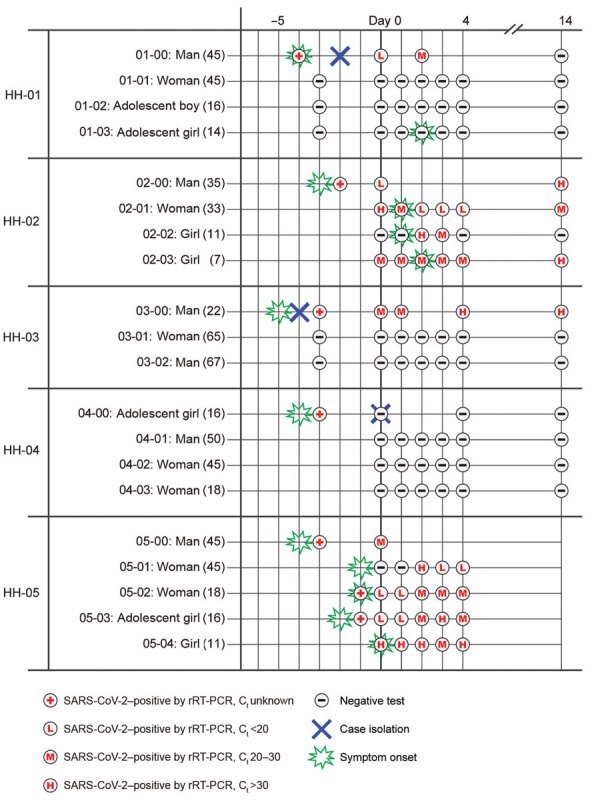
Results of rRT-PCR for SARS-CoV-2 and symptom onset among index case-patients, SARS-CoV-2–positive household contacts, and SARS-CoV-2–negative household contacts in study of initial virus shedding in SARS-CoV-2, Utah, USA, April–May 2020. The timelines of symptom onset and testing dates preceding and during the 15-day study period are ordered by individual households (HH-01–HH-05). Sex and age (in parentheses) are listed to the left. Symptom onset date is only included for household members who tested positive at any time during the study period or for whom onset of symptoms consistent with coronavirus disease prompted an interim visit from investigators. HH-05 opted out of day 14 nasopharyngeal specimen collection. C_t_, cycle threshold; HH, household; rRT-PCR, real-time reverse transcription PCR; SARS-CoV-2, severe acute respiratory syndrome coronavirus 2.

During April 19–25, 2020, a total of 5 households were enrolled, each consisting of an index case-patient and a median of 3 household members (range 2–4 persons). All index patients had the earliest symptom onset in their households. The day 0 visit occurred a median of 4 days (range 3–5 days) after symptom onset in the index patient. Secondary transmission was observed in 2 (40%) of the 5 households (HH-02 and HH-05), consisting of 7 (100%) of 7 contacts in these 2 households and 7 (47%) of 15 total household contacts in the study. The 8 contacts from the remaining 3 households did not become infected during the investigation (Figure 1). The median number of days between symptom onset in index patients and symptom onset in SARS-CoV-2–positive household contacts was 4 days (range 2–5 days). Eighty percent of index patients (4/5) were men and boys, and 80% of household contacts (12/15 [80%]) were women and girls (Table). The median age of index patients was 35 years (range 16–46 years). Of household contacts who tested positive, median age was 16 years (range 7–45 years); of household contacts who tested negative, median age was 45 years (range 14–67 years). Forty percent (2/5) of index patients, 43% (3/7) of SARS-CoV-2–positive household contacts, and 75% (6/8) of SARS-CoV-2–negative contacts reported >1 underlying medical condition.

**Table:** Characteristics and symptoms of index case-patients and household contacts testing positive or negative for severe acute respiratory syndrome coronavirus 2 by real-time reverse transcription PCR in study of initial virus shedding in SARS-CoV-2, Utah, USA, April–May 2020*

Characteristic	No. (%)		
	Index case-patients, n = 5	SARS-CoV-2–positive contacts, n = 7	SARS-CoV-2–negative contacts, n = 8
Age group, y			
<18	1 (20.0)	4 (57.1)	2 (25.0)
18–49	4 (80.0)	3 (42.9)	3 (37.5)
50–64	0	0	1 (12.5)
>65	0	0	2 (25.0)
Sex			
M	4 (80.0)	0	3 (37.5)
F	1 (20.0)	7 (100.0)	5 (62.5)
Race or ethnicity			
Non-Hispanic white	5 (100.0)	7 (100.0)	7 (87.5)
Hispanic	0	0	1 (12.5)
Underlying medical conditions			
Any underlying condition	2 (40.0)	3 (42.9)	6 (75.0)
Any chronic lung disease	1 (20.0)	0	2 (25.0)
Diabetes mellitus	0	1 (14.3)	0
Any cardiovascular disease	0	0	5 (62.5)
Any chronic renal disease	0	0	1 (12.5)
Any immunocompromised condition	1 (20.0)	0	1 (12.5)
Other chronic condition	1 (20.0)	2 (28.6)	1 (12.5)
No underlying medical condition	3 (60.0)	4 (57.1)	2 (25.0)
Smoking or vaping status			
Former history of smoking or vaping	1 (20.0)	0	1 (12.5)
Interactions with index case-patient			
Intimate physical contact	N/A	6 (85.7)	2 (25.0)
Close contact only	N/A	1 (14.3)	4 (50.0)
No interaction reported	N/A	0	2 (25.0)
Symptoms			
Any symptom	5 (100.0)	7 (100.0)	7 (87.5)
CSTE categories			
Classic†	4 (80.0)	5 (71.4)	2 (25.0)
Nonclassic‡	4 (80.0)	7 (100.0)	5 (62.5)
Asyndromic§	5 (100.0)	7 (100.0)	7 (87.5)
Other categories			
Neurologic¶	5 (100.0)	7 (100.0)	4 (50.0)
Lower respiratory#	4 (80.0)	6 (85.7)	2 (25.0)
Upper respiratory**	4 (80.0)	6 (85.7)	7 (87.5)
Constitutional††	4 (80.0)	7 (100.0)	5 (62.5)
Gastrointestinal‡‡	3 (60.0)	2 (28.6)	3 (37.5)

*CSTE, Council of State and Territorial Epidemiologists; SARS-CoV-2, severe acute respiratory syndrome coronavirus disease 2. †Cough, shortness of breath, or discomfort breathing.  ‡>2 of fever, myalgia, headache, chills, loss of taste or smell, or sore throat. §Any symptoms other than classic or nonclassic: fatigue, runny nose, nasal congestion, chest pain, wheezing, nausea or vomiting, or diarrhea. ¶Loss of taste (partial or complete), loss of smell (partial or complete), or headache. #Discomfort while breathing, wheezing, shortness of breath, chest pain, or cough (dry or productive). **Sore throat, nasal congestion, or runny nose. ††Chills, fever (measured or subjective), fatigue, or myalgia. ‡‡Abdominal pain, nausea or vomiting, or diarrhea.

Participants with a COVID-19 diagnosis had similar symptom profiles: headache was reported by 12/12 (100%); subjective fever, chills, fatigue, and nasal congestion were each reported by 10/12 (80%); myalgia was reported by 8/12 (67%); and partial loss of smell was reported by 7/12 (58%) (Appendix Figure). Classic symptoms were less common: dry cough was reported by 6/12 (50%); and productive cough, shortness of breath, and discomfort while breathing were each reported by <50% of those infected (Appendix Figure). Measured fever, sore throat, partial or full loss of taste, runny nose, chest pain, wheezing, nausea or vomiting, abdominal pain, and diarrhea were each reported by <33%. Nonclassic and asyndromic symptoms were also reported by SARS-CoV-2–negative household contacts (Appendix Figure). Median duration of illness was 7 days (range 2–14 days) among SARS-CoV-2–positive contacts and 11 days (range 4–19 days) among index case-patients. None of the 12 participants who tested positive for SARS-CoV-2 were hospitalized or experienced complications from pneumonia. Four (33%) of 12 tested positive on day 14, 3 (25%) were negative on day 14, and 5 (42%) refused swab tests on day 14. Among the 4 participants (02-00, 02-01, 02-03, and 03-00) with day 14 specimens positive for SARS-CoV-2 by rRT-PCR, 3 with C_t_ values <35 were cultured and viable virus was detected in 0/3 (0%). None of the 8 household members who tested negative by rRT-PCR tested positive by ELISA on day 0 or 14, suggesting no previous or undetected infections.

The 3 households (60%) that did not experience transmission (HH-01, HH-03, and HH-04) instituted household-level isolation practices. In HH-01, the index patient (01-00) moved out of the family home to a trailer on the property on the day of symptom onset (day –4), which coincided with the collection at a drive-through facility of the first specimen to test positive by rRT-PCR. He did report having had intimate contact (e.g., hugging or kissing) with 1 household member (01-01) after symptom onset but before diagnosis. The index patient wore gloves but no face mask on the few occasions he entered the family home. Household members also increased handwashing after diagnosis in the index patient. In HH-03, all household members had close contact (i.e., >10 minutes within 6 feet) with the index patient (03-00) between her symptom onset and the diagnosis; however, after diagnosis, the index patient used a separate bathroom (in addition to having her own bedroom) and ate meals separately from household contacts. Household contacts also increased disinfection of surfaces and handwashing after diagnosis in the index patient. In HH-04, between symptom onset and diagnosis in the index patient, 2 household contacts (04-02 and 04-03) had close contact with the index patient, and 1 contact (04-01) had intimate contact with the index patient. After diagnosis, the index patient stayed in a separate bedroom throughout the day (including for meals) but did not have access to a separate bathroom. He wore an N95 mask and gloves when leaving his room. Household members also disinfected surfaces regularly.

**Figure 2:**
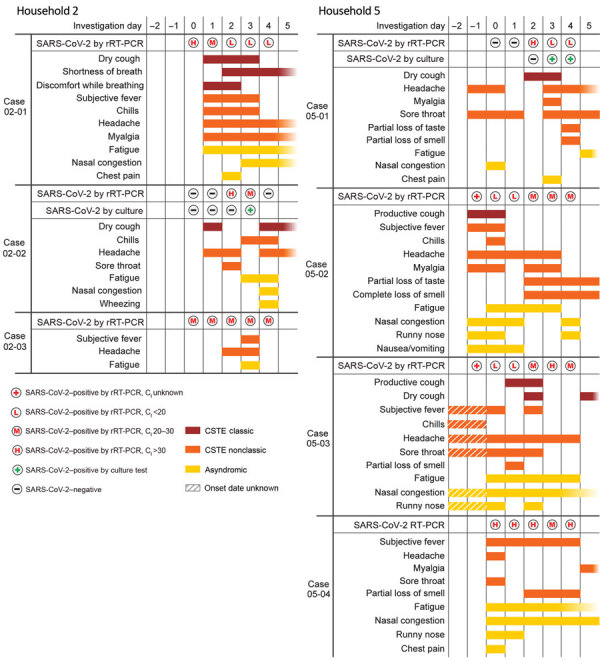
Symptom timing, symptom type, cycle threshold values, and viral culture results among household contacts positive for SARS-CoV-2 by rRT-PCR in study of initial virus shedding in SARS-CoV-2, Utah, USA, April–May 2020. The symptom onset and progression of 7 SARS-CoV-2–positive household contacts in households 2 and 5 (HH-02 and HH-05), who tested positive by real-time reverse transcription PCR, are detailed from first symptom onset to the end of the daily swabbing period (days 0–4). Fading bars indicate symptoms persisting after day 5. CSTE, Council of State and Territorial Epidemiologists; C_t_, cycle threshold; rRT-PCR, real-time reverse transcription PCR; SARS-CoV-2, severe acute respiratory syndrome coronavirus 2.

The 2 households (40%) where all contacts became infected (HH-02 and HH-05) did not institute household-level isolation practices, and all contacts had ongoing exposure to the index patient (Figure 2). During the investigation period, all members of both households were out of work and school because of school closures and stay-at-home recommendations in Salt Lake County. During the period from symptom onset in the index patient to enrollment in our study, all 7 (100%) contacts in these 2 households reported close contact with the index patient. During the same period, 6/7 (85%) household contacts who tested positive also reported intimate contact with the index patient after symptom onset, compared with 2/8 (25%) of household members who tested negative.

In HH-02, which consisted of a male index patient, his wife, and their 2 children, all 3 household contacts tested positive within 5 days of symptom onset in the index patient. Two of the contacts (02-01 and 02-03) shed virus while presymptomatic, and their symptoms did not occur until after their first SARS-CoV-2–positive test by rRT-PCR. The 33-year-old wife (02–01), who had ongoing exposure to the index patient for the duration of his illness, had C_t_ values that progressed from high (i.e., lower viral load) on her first positive test (day 0) to low (i.e., higher viral load) on her third test (day 2), when she first reported a combination of classic and nonclassic symptoms and fatigue. She remained SARS-CoV-2–positive by rRT-PCR at day 14, with a medium C_t_ value but no viable virus detected from culture. The second household member with presymptomatic virus shedding was a 7-year-old girl (02-03) whose daily C_t_ values were consistently medium during days 0–4. After testing positive for 2 days (days 0–1), she first reported nonclassic symptoms on day 2 and was symptomatic for only 2 days. She also remained positive at day 14, with a high C_t_ value and no viable virus detected from culture. The third household member, an 11-year-old girl (02-02), converted to rRT-PCR–positive (day 2) after testing negative for 2 days (days 0–1). She reported classic and nonclassic symptoms (dry cough and headache) on day 1. On day 2, she tested positive with a high C_t_ value and reported onset of a sore throat. On day 3, she tested positive with a medium C_t_ value, reported onset of chills and fatigue, and had a positive viral culture, before testing negative again on day 4.

Household 5 (HH-05) consisted of a male index patient, his wife, an adult child, and 2 adolescent children. All 4 household contacts tested positive for SARS-CoV-2 by rRT-PCR within 6 days of symptom onset in the index patient. Although all household contacts sought drive-through testing the day before the investigation began (day –1), only the 18-year-old woman (05-02) and the 16-year-old girl (05-03) met symptom criteria for testing; consequently, both had 1 positive test result before the investigation. The 16-year-old girl (05-03) reported nonclassic and asyndromic symptoms (day –2) starting the day before her first positive test by rRT-PCR (day –1) administered at the drive-through facility. Her next 2 positive tests, administered by the investigation team on day 0 and day 1, had low Ct values and coincided with the onset of fatigue (day 0) and cough (day 1). The 18-year-old woman (05-02) and 11-year-old girl (05-04) each reported symptoms starting the same day as their first rRT-PCR–positive tests, with 1 (05-02) administered at a drive-through facility (day –1) and the other (05-04) by the investigation team (day 0). Although they had a range of nonclassic and asyndromic symptoms during illness, the 18-year-old female (05-02) had a cough at onset (day –1) and low C_t_ values for her first 2 team-administered tests (days 0–1), whereas the 11-year-old girl had generally milder illness and high C_t_ values (i.e., lower viral load) for 4 of 5 tests. The 45-year-old woman (05-01) tested negative for 2 days (days 0–1) and had nonclassic and asyndromic symptoms for 3 days (days –1 to 1) before her first positive test on day 2; on that day, she tested positive with a high C_t_ value and reported onset of a cough. Her next 2 positive tests (days 3–4) had low C_t_ values, coinciding with onset of additional symptoms (chest pain, myalgia, and loss of taste and smell) and positive viral cultures on both days. All HH-05 members refused testing by nasopharyngeal swab on day 14 because of concerns about the potential need to self-isolate beyond 14 days after an initial positive test, which was the required isolation period at the time in Salt Lake County.

## Discussion

In our study, we found that symptoms of secondary SARS-CoV-2 infection occurred in 7 household contacts of index COVID-19 patients starting <2 days before and <3 days after the observed initiation of viral shedding. The median interval of 4 days between symptom onset in index patients and symptom onset in their respective SARS-CoV-2–positive household contacts was similar to that reported in other household studies (*10**,**11*,*15*). Timely enrollment in our investigation (median 4 days after symptom onset in the index patient), however, allowed us to observe the timing and characteristics of initial viral shedding with a level of granularity not attained in previous studies.

For the household members (02-02 and 05-01) in whom we observed the initiation of viral shedding (i.e., SARS-CoV-2–positive result by rRT-PCR after a negative test), the first day of shedding corresponded with a high C_t_ value, and the second day of shedding corresponded with a lower C_t_ value, a positive viral culture, and the onset of new symptoms. These observations suggest that although the initiation of shedding marks the beginning of potential infectiousness, higher likelihood of virus transmission (indicated by positive viral culture) might coincide with lower C_t_ values and the appearance of additional symptoms (*16*). Although 4 persons continued shedding virus >12 days after onset of symptoms, no culturable and potentially infectious virus could be isolated from the specimens collected.

For the 2 household members (02-01 and 02-03) in whom we observed presymptomatic viral shedding, initial shedding corresponded with medium or high C_t_ values and occurred for 1–2 days before symptom onset. In 1 patient (02-01), the onset of symptoms coincided with a progression from high to medium C_t_ value, and new, additional symptoms coincided with further progression from medium to low C_t_ values. These findings mirror previous observations of presymptomatic shedding but suggest that viral load might increase as symptoms appear or progress. Among all SARS-CoV-2–positive contacts, symptoms were generally mild and sometimes transient. Of note, only 4 of 7 cases reported classic lower respiratory symptoms. In HH-02, the 2 contacts (02-01 and 02-02) who reported lower respiratory symptoms had them at illness onset, alongside several other symptoms. In HH-05, of the 3 contacts who had lower respiratory symptoms (05-01, 05-02, 05-03), two (05-01 and 05-03) reported them several days after symptom onset. Reports of symptoms by household contacts who remained SARS-CoV-2–negative could suggest other viral illnesses, allergies, underlying medical conditions, or stress-related effects of living with a person with COVID-19 (*17*).

Our findings suggest that household-level isolation practices could have been effective in preventing transmission. Findings from the 2003 SARS-CoV-1 epidemic showed that isolation of a patient before peak shedding was effective in reducing household transmission (*18*), and our results suggest that adopting precautionary measures can be effective in preventing secondary household transmission. In the households where no transmission was experienced, providing an index patient with separate sleeping quarters and avoiding face-to-face interactions (e.g., shared mealtimes) appeared sufficient to prevent transmission, even in households where close or intimate contact had occurred before diagnosis. Our findings show, however, that some persons infected with SARS-CoV-2 could begin shedding virus before being prompted to isolate by the onset of symptoms. In contrast to the households with no transmission, which consisted primarily of adults, the 2 households with secondary transmission to all contacts consisted of parents and their adolescent or preadolescent children. In these households, childcare needs and difficulties maintaining full isolation caused members to eschew precautionary practices, particularly after other household members were known to be infected.

Our study has some limitations. First, our household case-series was small because of the intensive nature of our early monitoring protocol; it was also biased toward index patients who were sufficiently symptomatic to be tested but whose disease was not severe enough to require hospitalization. Second, although all SARS-CoV-2–positive contacts had symptom onset >2 days (the estimated minimum incubation period) after the corresponding index patient, we cannot rule out the possibility of transmission from 1 presymptomatic household contact to another contact. Finally, symptom data relied on self-reporting, and symptoms might have been present before or after they were reported by patients. Three (20%) of 15 household contacts were children <13 years of age, who might have had more difficulty recognizing and reporting symptoms. Patient subjectivity could contribute to whether virus shedding or symptom onset is observed first.

In conclusion, our findings indicate that shedding of the SARS-CoV-2 virus might occur early in the disease course before symptom onset and clinical diagnosis, or it could occur when symptoms are mild or even absent. Persons with confirmed COVID-19 or who have had close contact with someone with confirmed COVID-19 should limit close contact with others, including household members, for 14 days. Persons who have been exposed to SARS-CoV-2 should be vigilant to the onset of mild symptoms; if they have not already limited close contact with household members or other persons, the onset of even mild symptoms should prompt additional caution and efforts to limit close contact. In addition, wearing masks or cloth face covers, practicing hand hygiene, and disinfecting surfaces regularly might reduce risk for transmission in households (*19*). Stay-at-home orders and at-home self-treatment of COVID-19 in the United States requires clear communication of such guidelines to prevent household transmission.

Appendix Additional information about characteristics and timing of initial virus shedding in severe acute respiratory syndrome coronavirus 2, Utah, USA.
