# Supplementary material for: Characteristics and Timing of Initial Virus Shedding in Severe Acute Respiratory Syndrome Coronavirus 2, Utah, USA
Source: Emerg Infect Dis. 2021 Feb;27(2):352–9. doi: 10.3201/eid2702.203517 (PMC7853554; doi:10.3201/eid2702.203517)
Supplement: Appendix — Additional information about characteristics and timing of initial virus shedding in severe acute respiratory syndrome coronavirus 2, Utah, USA. [file 20-3517-Techapp-s1.pdf]

Article DOI: https://doi.org/10.3201/eid2702.203517

Characteristics and Timing of Initial Virus Shedding in Severe Acute Respiratory Syndrome Coronavirus 2, Utah, USA

Appendix

Appendix Table. Symptom duration among index case-patients, severe acute respiratory syndrome coronavirus 2–positive and –negative household contacts, Utah, April–May 2020*

	Index case-patients, n = 5		SARS-CoV-2–positive contacts, n = 7		SARS-CoV-2–negative contacts, n = 8	
Symptom type	No. (%)	Median duration, d (range)	No. (%)	Median duration, d (range)	No. (%)	Median duration, d (range)
Overall illness	5 (100.0)	11 (4–19)	7 (100.0)	7 (2–14)	7 (87.5)	12 (2–25)
Gastrointestinal	3 (60.0)	2 (2–13)	2 (28.6)	1.5 (1–2)	3 (37.5)	1 (1–5)
Abdominal pain	2 (40.0)	2 (2–2)	1 (14.3)	1 (1–1)	2 (25.0)	1.5 (1–2)
Nausea/vomiting	1 (20.0)	2 (2–2)	1 (14.3)	2 (2–2)	1 (12.5)	1 (1–1)
Diarrhea	2 (40.0)	6 (2–10)	0 (0)	N/A	1 (12.5)	1 (1–1)
Neurologic	5 (100.0)	5 (1–11)	7 (100.0)	6 (2–12)	4 (50.0)	4 (1–12)
Loss of taste (partial or complete)	1 (20.0)	1 (1–1)	2 (28.6)	5 (4–6)	0 (0)	N/A
Loss of smell (partial or complete)	4 (80.0)	2 (1–3)	4 (57.1)	3.5 (1–6)	0 (0)	N/A
Headache	5 (100.0)	4 (1–7)	7 (100.0)	5 (1–9)	4 (50.0)	3 (1–7)
Lower respiratory	4 (80.0)	4 (1–15)	6 (85.7)	4 (1–6)	2 (25.0)	9 (3–15)
Discomfort while breathing	2 (40.0)	8 (1–15)	1 (14.3)	2 (2–2)	1 (12.5)	1 (1–1)
Wheezing	1 (20.0)	1 (1–1)	1 (14.3)	1 (1–1)	1 (12.5)	13 (13–13)
Shortness of breath	2 (40.0)	4 (3–5)	1 (14.3)	5 (5–5)	2 (25.0)	4.5 (1–8)
Chest pain	2 (40.0)	8.5 (2–15)	3 (42.9)	1 (1–1)	1 (12.5)	2 (2–2)
Cough (dry or productive)	3 (60.0)	2 (1–4)	5 (71.4)	4 (1–4)	1 (12.5)	3 (3–3)
Upper respiratory	4 (80.0)	4 (1–7)	6 (85.7)	9 (3–13)	7 (87.5)	4 (1–15)
Sore throat	0 (0)	N/A	4 (57.1)	2 (1–5)	5 (62.5)	2 (1–6)
Nasal congestion	4 (80.0)	2.5 (1–7)	6 (85.7)	6.5 (1–11)	5 (62.5)	2 (1–10)
Runny nose	2 (40.0)	5.5 (4–7)	3 (42.9)	2 (2–8)	4 (50.0)	2.5 (1–4)
Constitutional	4 (80.0)	6 (1–14)	7 (100.0)	5 (1–12)	5 (62.5)	5 (2–15)
Chills	3 (60.0)	2 (1–2)	4 (57.1)	2 (1–3)	1 (12.5)	1 (1–1)
Fever (measured or subjective)	3 (60.0)	2 (1–2)	5 (71.4)	2 (1–5)	2 (25.0)	1 (1–1)
Fatigue	3 (60.0)	4 (1–5)	7 (100.0)	5 (1–10)	5 (62.5)	5 (1–14)
Myalgia	3 (60.0)	2 (1–4)	4 (57.1)	3 (1–6)	1 (12.5)	5 (5–5)
CSTE symptom classification						
Classic†	4 (80.0)	4 (1–15)	5 (71.4)	6 (1–6)	2 (25.0)	9 (3–15)
Nonclassic‡	4 (80.0)	6.5 (1–11)	7 (100.0)	6 (2–12)	5 (62.5)	5 (1–12)
Asyndromic§	5 (100.0)	7 (1–15)	7 (100.0)	8 (1–13)	7 (87.5)	8 (1–15)

*SARS-CoV-2, severe acute respiratory syndrome coronavirus 2; CSTE, Council of State and Territorial Epidemiologists.
†Cough, shortness of breath, or discomfort breathing.
‡≥2 of fever, myalgia, headache, chills, loss of taste or smell, or sore throat.
§Any symptoms other than classic or nonclassic: fatigue, runny nose, nasal congestion, chest pain, wheezing, nausea/vomiting, or diarrhea.

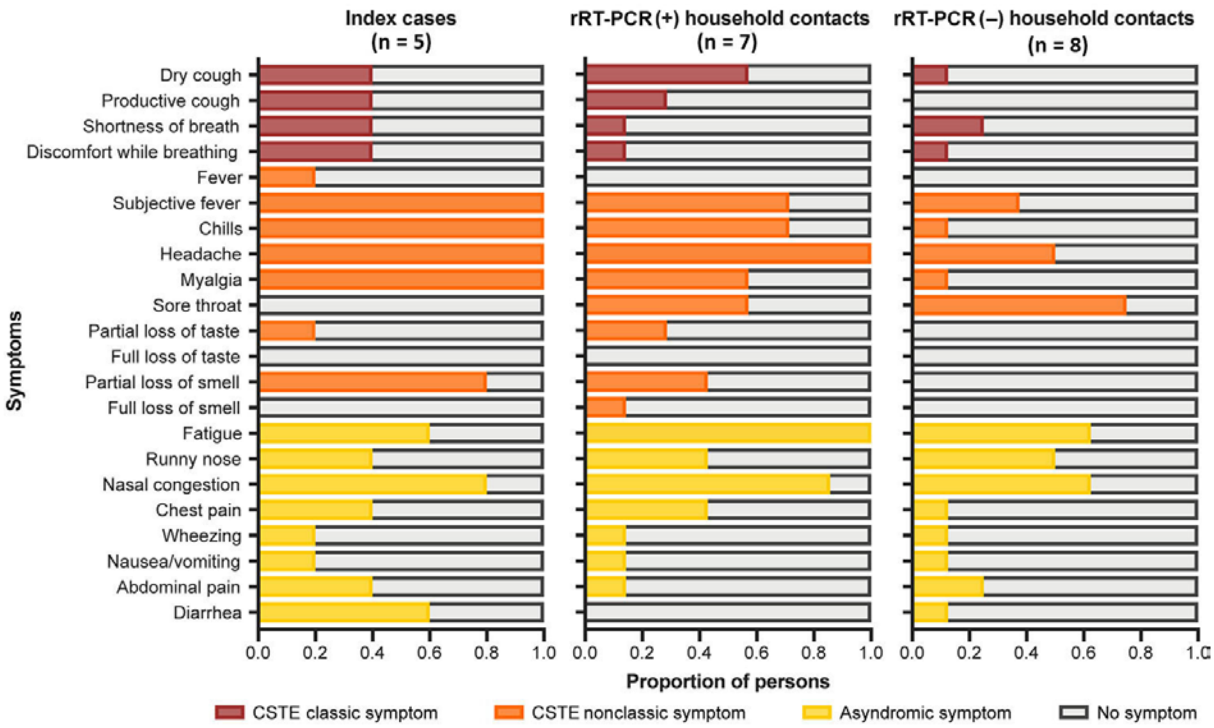

Appendix Figure. Symptoms experienced among index case-patients and household contacts who tested positive or negative for severe acute respiratory syndrome coronavirus 2 (SARS-CoV-2) by rRT-PCR, Utah, April–May 2020. Participants, including index case-patients and household contacts, were asked to report any symptoms they experienced since symptom onset in each household's index patient and during the 15-day study period. The proportion of persons who experienced a specific symptom is shown by index case-patients, SARS-CoV-2–positive household contacts, and SARS-CoV-2–negative household contacts. CSTE, Council of State and Territorial Epidemiologists; rRT-PCR, real-time reverse transcription PCR.
